# Immunogenicity and safety of a second booster dose of an acellular pertussis vaccine combined with reduced antigen content diphtheria-tetanus toxoids 10 years after a first booster in adolescence: An open, phase III, non-randomized, multi-center study

**DOI:** 10.1080/21645515.2018.1460292

**Published:** 2018-05-10

**Authors:** Martina Kovac, Lusiné Kostanyan, Narcisa Mesaros, Sherine Kuriyakose, Meera Varman

**Affiliations:** a GSK, Vaccines, Wavre, Belgium; b XPE Pharma & Science, Wavre, Belgium, Belgium; c GSK Vaccines, Bangalore, India; d Pediatric Infectious Disease, Creighton University, Omaha, NE, United States

**Keywords:** decennial booster, diphtheria, pertussis, tetanus, vaccination

## Abstract

Pertussis is a highly contagious disease, for which periodic peaks in incidence and an increasing number of outbreaks have been observed over the last decades. The reduced-antigen-content tetanus-diphtheria-acellular pertussis vaccine (Tdap) can be used to boost individuals aged ≥10 years, vaccinated in infancy with a diphtheria-tetanus-acellular pertussis vaccine (DTaP), to reduce pertussis morbidity and maintain protection against diphtheria and tetanus throughout adolescence and adulthood. This phase III, open-label, non-randomized, multicenter follow-up study (NCT01738477) enrolled 19–30-year-old participants from the United States who had received booster vaccination 10 years earlier with either Tdap (Tdap group) or Td (Td group). In total, 128 (Tdap group) and 37 (Td group) participants received Tdap vaccination. After administration of Tdap, all participants were seroprotected (antibody concentrations ≥0.1 international units [IU]/ml) against diphtheria and tetanus. Immune responses to a second Tdap dose in the Tdap group were shown to be non-inferior to responses elicited by a first Tdap dose in the Td group for diphtheria and tetanus and to a 3-dose DTaP vaccination during infancy for pertussis antigens (primary objectives). Post-booster vaccination, all participants in both groups had antibody concentrations above assay cut-offs and antibody geometric mean concentrations increased by 3.8–15.5-fold compared to pre-booster levels for all antigens. The incidence of adverse events was similar in the Td (80.6%) and Tdap (85.6%) groups (no serious adverse events reported). A Tdap dose administered after previous Td or Tdap vaccination was shown to be immunogenic and well-tolerated in young adults, supporting repeated vaccination with Tdap at 10-year intervals.

## Introduction

Pertussis or whooping cough, a highly contagious respiratory tract infection caused by *Bordetella pertussis*, is rapidly transferred via respiratory droplets and manifests as severe coughing.^1^ Despite the use of vaccination worldwide, the incidence of pertussis remains relatively high, with 139,535 cases reported globally in 2016^2^ and peaks occurring every 3–5 years.^3^

In the United States (US), 32,971 confirmed pertussis cases were reported in 2014, an increase of 15% compared with the previous year;^4^ however, the number of cases dropped to 15,737 in 2016.^5^ Overall, resurgence of pertussis has been broadly recognized.^6-10^ In addition to the already known high burden of disease in infants too young to be protected by vaccination, pertussis incidence rates have also increased in adolescents and adults, even in countries with high vaccination coverages during childhood.^1^ Recent data show that adolescents are the most affected age group after infants, with a reported age-specific incidence of pertussis of 13.9/100,000 in the 11–19 year age group in the US in 2016^5^ and 23.6/100,000 in 10–14-year-olds in European countries in 2015.^11^ The cause of periodic outbreaks, which follow the natural cycle of disease, is multifaceted.^12^^,^^13^ Immunity following both natural infection and vaccination is short-lived, likely due in part to pathogenic evolution and, although pertussis vaccines reduce disease severity, they might be more limited in preventing transmission than previously anticipated.^12^^,^^14^^,^^15^ Following the switch from whole-cellular to acellular pertussis vaccines due to safety concerns,^16^ an increase in pertussis infections has been reported in several countries,^6-10^ as the antibody persistence after acellular versus whole-cell pertussis vaccination seems to be more prone to decline.^17^

In the US, the diphtheria-tetanus-acellular pertussis vaccine (DTaP) is recommended for infants and young children, and since 2005, reduced antigen content diphtheria-tetanus-acellular pertussis vaccine (Tdap) products are approved for use predominantly for boosting adolescents and adults.^18^^,^^19^ The Advisory Committee on Immunization Practices currently recommends administration of a single Tdap dose (instead of tetanus-diphtheria vaccine [Td]) to adolescents aged 11–18 years, to all adolescents and adults who have not received Tdap previously, regardless of the interval since the last Td dose,^20^ to all unvaccinated ≥65-year-olds who anticipate close contact with an infant aged <12 months, during every pregnancy at 27–36 weeks of gestation (preferably during the early part of this interval) and to postpartum mothers who were not vaccinated during pregnancy.^21^ Repeated decennial booster doses throughout life are also currently recommended for immunization against diphtheria and tetanus, but not against pertussis.^19^

Recent studies attest waning immunity to pertussis approximately 5–10 years following childhood vaccination.^18^^,^^22^^,^^23^ A decline in Tdap protection in adolescents within 2–4 years following vaccination was observed, together with a causal relationship between the lack of long-term protection and increased pertussis emergences.^24^ Several US studies also showed that Tdap vaccination in adolescents failed to prevent pertussis outbreaks and achieved only a moderate protection in the first year with little protection remaining 4 years post vaccination.^25^^,^^26^ In Australia and Finland, antibody persistence against diphtheria, tetanus and pertussis has been evaluated in participants who received a second dose of Tdap, 10 years following a first booster dose.^27^^,^^28^ By this time, antibody concentrations had nearly dropped to pre-booster vaccination levels, but a second Tdap dose triggered a robust immune response against all antigens, with a frequency of adverse events (AEs) in the expected limits, irrespective of the vaccination history.^27^^,^^28^

Although waning of post-vaccination antibody levels against pertussis warrants the administration of an additional booster vaccination in adolescents and adults, the immunogenicity and safety data for a second Tdap booster dose are lacking in the US.

We previously assessed the immunogenicity and safety of Tdap (*Boostrix*, GSK) compared to a control Td vaccine (MassBiologics) administered as booster vaccination in 10–18-year-olds who had received primary immunization with whole-cell or acellular DTP vaccines.^29^ In this follow-up study, we evaluate the non-inferiority of a second Tdap booster dose with respect to immune responses to diphtheria and tetanus when compared with that of a first dose in participants having received a Td booster dose 10 years earlier and to pertussis antigens when compared with a 3 dose-series of a DTaP vaccine (*Infanrix*, GSK) in infants who received this vaccine in a German household contact efficacy study.^30^ Antibody persistence for each vaccine antigen at 10 years following the initial Td/Tdap booster vaccination, and safety and immunogenicity of the new booster dose were also assessed.

## Results

### Demographic characteristics

**Figure 1.**
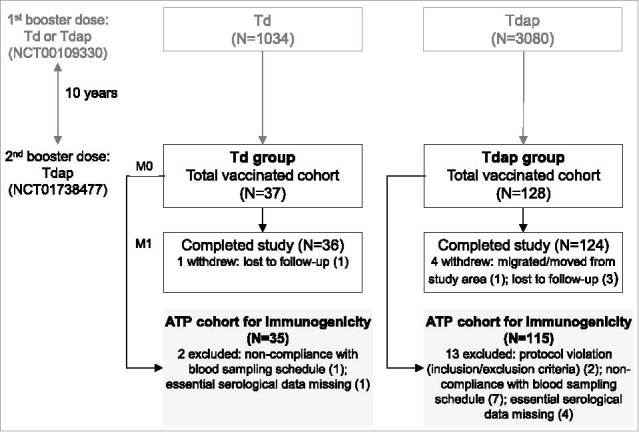
Participant flowchart. Footnote: Td group, participants receiving Td as first booster dose in the primary study and Tdap as decennial booster dose (second booster dose) in the current study; Tdap group, participants receiving Tdap booster doses 10 years apart; N, number of participants in each group; M, month; ATP, according-to-protocol.

A total of 165 individuals were vaccinated with Tdap during the present follow-up study, 37 having previously received Td as a first booster dose (Td group) and 128 having previously received a first Tdap booster dose (Tdap group); 160 participants completed the study. The according-to-protocol (ATP) cohort for immunogenicity included 150 participants (Fig. 1). Overall, 54.5% of participants were male and the majority (87.9%) was White/Caucasian. Demographic characteristics at enrolment were balanced between groups (Table 1).

**Table 1.** Summary of demographic characteristics (total vaccinated cohort).

	Td group	Tdap group	Total
	N = 37	N = 128	N = 165
Mean age ± SD, years	23.3 ± 2.4	23.5 ± 2.1	23.5 ± 2.1
Median age (minimum–maximum), years	23 (20–29)	23 (20–29)	23 (20–29)
Gender			
Male, n (%)	19 (51.4)	71 (55.5)	90 (54.5)
Female	18 (48.6)	57 (44.5)	75 (45.5)
Ethnicity, n (%)			
American Hispanic or Latino	4 (10.8)	12 (9.4)	16 (9.7)
Not American Hispanic or Latino	33 (89.2)	116 (90.6)	149 (90.3)
Geographic ancestry, n (%)			
Black	1 (2.7)	6 (4.7)	7 (4.2)
White/Caucasian	31 (83.8)	114 (89.1)	145 (87.9)
Oriental	0 (0.0)	1 (0.8)	1 (0.6)
Other	5 (13.5)	7 (5.5)	12 (7.3)

Td group, participants receiving Td as first booster dose in the primary study and Tdap as decennial booster dose (second booster dose) in the currentstudy;Tdapgroup, participants receiving Tdap booster doses 10 years apart; N, number of participants; n (%), number (percentage) of participants in each category; SD, standard deviation.

### Immunogenicity

Both pre-defined co-primary non-inferiority objectives were met. The immune responses to diphtheria and tetanus elicited by a second Tdap dose were shown to be non-inferior to a first Tdap dose following previous Td booster immunization (first co-primary objective), as the lower limits (LLs) of the 95% confidence intervals (CIs) on the between-group difference for the percentage of participants seroprotected against diphtheria and tetanus (*i.e*, with antibody concentrations ≥0.1 International Units [IU]/ml) were above the pre-specified non-inferiority margin of -10% (Table 2).

**Table 2.** Results of co-primary objectives.

*Response to diphtheria and tetanus antigens (according-to-protocol cohort for immunogenicity)*					
	Td group		Tdap group		Difference in seroprotection rates
	N	n (%)	N	n (%)	Tdap minus Td, % (95% CI)
Diphtheria	35	35 (100)	115	115 (100)	0.00 (**−3.25**–9.95)
Tetanus	35	35 (100)	115	115 (100)	0.00 (**−3.25**–9.95)
*Response to pertussis antigens (total vaccinated cohort)*					
	Comparator group^a^		Tdap group		Tdap/ Comparator
	N	GMC	N	GMC	GMC ratio (95% CI)
Pertussis toxoid	2884	41.7	124	83.5	2.00 (**1.69**–2.37)
Filamentous hemagglutinin	685	47.2	124	285.5	6.05 (**5.14**–7.11)
Pertactin	631	113.0	124	442.6	3.92 (**3.22**–4.76)

Td group, participants receiving Td as first booster dose in the primary study and Tdap as decennial booster dose (second booster dose) in the current study; Tdap group, participants receiving Tdap booster doses 10 years apart; N, number of participants with available results; n (%), number (percentage) of seroprotected participants (with anti-diphteria/tetanus antibody concentrations ≥0.1 IU/ml); 95% CI, 95% standardized asymptotic confidence interval; GMC, geometric mean antibody concentration.

Note:

a Infants vaccinated with DTaP in a German efficacy study.^30^ Given the absence of serologic correlates of protection against pertussis, an immuno-bridging approach was used to assess immune responses to pertussis antigens, by extrapolating the efficacy of a vaccine against pertussis as demonstrated in infants to an older age group, as previously described.^29^

Values above the non-inferiority margin (the lower limit of the 95% CI ≥-10% for the first co-primary objective and ≥0.67 for the second co-primary objective) are bolded.

The immune responses to pertussis antigens elicited by a second decennial Tdap booster dose were shown to be non-inferior to those induced by a 3-dose DTaP series administered during infancy (second co-primary objective). The LLs of the 95% CI on the anti-pertussis toxoid (PT), anti-filamentous hemagglutinin (FHA) and anti-pertactin (PRN) antibody geometric mean concentration (GMC) ratios (Tdap group over the group receiving DTaP in the German household efficacy study) were ≥0.67 (Table 2).

Ten years after the first Tdap or Td dose and before vaccination with the second booster dose, seroprotection rates and anti-diphtheria and anti-tetanus antibody levels were within similar ranges (Table 3). One month following the Tdap dose, all participants in both groups had antibody concentrations ≥0.1 IU/ml and ≥97.1% had antibody concentrations ≥1.0 IU/ml against diphtheria and tetanus (Table 3). Antibody GMCs were comparable between groups (Table 3), showing increases of 3.8–5.5-fold from pre-booster vaccination values.

**Table 3.** Immune responses to Tdap vaccination (according-to-protocol cohort for immunogenicity).

		Td group (N = 35)		Tdap group (N = 115)		
		Threshold (IU/ml)	% (95% CI)	GMC (95% CI)	% (95% CI)	GMC (95% CI)	
*Diphtheria*						
Pre-booster	≥0.1	100 (90.0–100)	1.6 (1.1–2.3)	100 (96.8–100)	1.6 (1.3–2.1)	
	≥1	65.7 (47.8–80.9)		60.9 (51.3–69.8)		
Post-booster	≥0.1	100 (90.0–100)	6.8 (5.4–8.6)	100 (96.8–100)	6.0 (5.3–6.9)	
	≥1	97.1 (85.1–99.9)		100 (96.8–100)		
*Tetanus*						
Pre-booster	≥0.1	100 (90.0–100)	1.8 (1.4–2.4)	100 (96.8–100)	1.8 (1.5–2.2)	
	≥1	77.1 (59.9–89.6)		74.8 (65.8–82.4)		
Post-booster	≥0.1	100 (90.0–100)	9.9 (7.9–12.5)	100 (96.8–100)	9.7 (8.5–11.0)	
	≥1	100 (90.0–100)		100 (96.8–100)		
*PT*						
Pre-booster	≥2.693	60.0 (42.1–76.1)	5.3 (3.4–8.2)	87.8 (80.4–93.2)	9.9 (8.1–12.2)	
Post-booster		100 (90.0–100)	66.2 (50.8–86.2)	100 (96.8–100)	87.3 (74.5–102.4)	
*FHA*						
Pre-booster	≥2.046	97.1 (85.1–99.9)	21.7 (13.4–35.4)	100 (96.8–100)	36.9 (31.5–43.3)	
Post-booster		100 (90.0–100)	336.2 (250.0–452.2)	100 (96.8–100)	290.5 (252.5–334.2)	
*PRN*						
Pre-booster	≥2.187	94.3 (80.8–99.3)	27.8 (13.7–56.3)	100 (96.8–100)	71.6 (56.7–90.6)	
Post-booster		100 (90.0–100)	425.5 (281.9–642.3)	100 (96.8–100)	463.3 (390.8–549.3)	

Td group, participants receiving Td as first booster dose in the primary study and Tdap as decennial booster dose (second booster dose) in the current study; Tdap group, participants receiving Tdap booster doses 10 years apart; N, number of participants with available results; IU, international units; %, percentage of participants with antibody concentrations at least the pre-specified thresholds; CI, confidence interval; GMC, geometric mean antibody concentration; Pre-booster, before vaccination in the current study; Post-booster, 1 month post-booster vaccination in the current study; PT, pertussis toxoid; FHA, filamentous hemagglutinin; PRN, pertactin.

Pre-booster vaccination, the percentage of seropositive participants (*i.e.*, with antibody concentrations above the assay cut-offs) for PT was significantly higher in the Tdap group (87.8%; 95% CI: 80.4–93.2%) than in the Td group (60.0%; 95% CI: 42.1–76.1%). For the other pertussis antigens, the percentages of initially seropositive participants were similar between the two groups, with ≥97.1% and ≥94.3% having concentrations higher than the assay cut-off for FHA and PRN, respectively. Following vaccination with Tdap, all participants in both groups were seropositive for each of the three pertussis antigens (Table 3). Antibody GMCs for all pertussis components were comparable between the Td and Tdap groups (Table 3) and increased 6.5–15.5-fold from pre-vaccination levels.

Around 40.0% of participants showed a booster response to diphtheria and ≤60.0% to tetanus antigens in both groups. When excluding participants with pre-vaccination anti-diphtheria and anti-tetanus antibody concentrations ≥6 IU/ml, 70.0% and 59.2% demonstrated a booster response to diphtheria and 84.4% and 82.9% to tetanus in the Td and Tdap groups, respectively (Table 4). Booster responses to pertussis antigens also varied slightly with pre-vaccination levels, but ≥90.4% of participants demonstrated a booster response to PT and FHA and ≥68.7% to PRN, regardless of baseline serostatus in both groups (Table 4).

**Table 4.** Booster response and alternative booster response to diphtheria and tetanus and booster response to pertussis components, overall and by initial serostatus (according-to-protocol cohort for immunogenicity).

	Td group		Tdap group	
	N	% (95% CI)	N	% (95% CI)
*Diphtheria*				
Booster response	35	40.0 (23.9–57.9)	115	40.9 (31.8–50.4)
Alternative booster response	30	70.0 (50.6–85.3)	98	59.2 (48.8–69.0)
*Tetanus*				
Booster response	35	60.0 (42.1–76.1)	115	55.7 (46.1–64.9)
Alternative booster response	32	84.4 (67.2–94.7)	105	82.9 (74.3–89.5)
Pre-vaccination status				
*PT*				
S−	14	100 (76.8–100)	14	85.7 (57.2–98.2)
S+ (<4*2.693 IU/mL)	9	88.9 (51.8–99.7)	47	95.7 (85.5–99.5)
S+ (≥4*2.693 IU/mL)	12	91.7 (61.5–99.8)	54	90.7 (79.7–96.9)
Overall	35	94.3 (80.8–99.3)	115	92.2 (85.7–96.4)
*FHA*				
S−	1	100 (2.5–100)	0	—
S+ (<4*2.046 IU/mL)	8	100 (63.1–100)	4	100 (39.8–100)
S+ (≥4*2.046 IU/mL)	26	96.2 (80.4–99.9)	111	90.1 (83.0–94.9)
Overall	35	97.1 (85.1–99.9)	115	90.4 (83.5–95.1)
*PRN*				
S−	2	100 (15.8–100)	0	—
S+ (<4*2.187 IU/mL)	9	100 (66.4–100)	9	100 (66.4–100)
S+ (≥4*2.187 IU/mL)	24	75.0 (53.3–90.2)	106	66.0 (56.2–75.0)
Overall	35	82.9 (66.4–93.4)	115	68.7 (59.4–77.0)

Td group, participants receiving Td as first booster dose in the primary study and Tdap as decennial booster dose (second booster dose) inthecurrentstudy;Tdapgroup, participants receiving Tdap booster doses 10 years apart; N,numberofparticipants with available results; %, percentage of participants with booster response; CI, confidenceinterval;S-,seronegative;S+, seropositive;Overall,participantseitherseropositive or seronegative at pre-vaccination; N, number of participants with both pre- and post-vaccination results available; PT, pertussis toxoid; FHA, filamentous hemagglutinin; PRN, pertactin; IU, international units.

Note:Booster responsesweredefined onemonthpost-vaccinationwithTdapas follows: (i) for diphtheria and tetanus, antibody concentrations ≥0.4 IU/ml in initiallyseronegativeparticipants(i.e.,withpre-vaccinationantibodyconcentrations <0.1 IU/ml) or as a ≥4-fold increaseofantibodyconcentrations ininitially seropositive participants (i.e., with pre-vaccination antibody concentrations ≥ 0.1 IU/ml);(ii)foreachpertussiscomponent,as anincreaseof antibody concentrations of ≥4*assay cut-off in initially seronegative participants (i.e., with pre-vaccinationantibodyconcentrationsbelowtheassaycut-off),asa≥4-foldincreasefrom pre-boosterlevelsininitiallyseropositiveparticipants with concentrations <4*assaycut-off,andasa≥2-foldincreasefrompre-booster levels in initially seropositive participants with concentrations ≥ 4*assay cut-off.

Alternative booster response for diphtheria and tetanus excluded participants with pre-vaccination antibody concentration ≥ 6.0 IU/ml and was defined one month post-vaccinationwithTdapas:(i)antibodyconcentrations ≥0.4 IU/ml in participantswithpre-vaccinationconcentrations<0.1IU/ml;(ii) a≥4-fold increase from pre-booster levels of antibody concentrations in participants with pre-vaccinationconcentrations<1.0 IU/ml;(iii) ≥2-fold increase from pre-booster levels ofantibodyconcentrationsinparticipantswithpre-vaccinationconcentrations between ≥1.0 and < 6.0 IU/ml.

In a sensitivity analysis performed in the ATP cohort for immunogenicity at each timepoint, no impact related to drop-out was observed on immunogenicity results (see Online Supplement).

### Safety

During the 4-day follow-up period, any solicited and unsolicited AE was reported for 80.6% of participants in the Td group and 85.6% in the Tdap group. Pain was the most frequently reported solicited local AE, by 58.3% and 77.6% of participants in the Td and Tdap groups, respectively. Grade 3 pain was reported by no more than 5.6% of participants in each group (Table 5). Fatigue (reported for 22.2% of participants in the Td group and 30.4% in Tdap group) and headache (reported for 22.2% of adults in the Td group and 32.0% of those in the Tdap group) were the most frequently reported solicited general AEs. Grade 3 general AEs were recorded in ≤2.4% of participants in each group (Table 5).

**Table 5.** Summary of reactogenicity and safety following administration of a Tdap dose (total vaccinated cohort).

	Td group		Tdap group	
	n	% (95% CI)	n	% (95% CI)
	N = 36		N = 125	
Solicited adverse events				
*Solicited local* adverse events				
Pain	21	58.3 (40.8–74.5)	97	77.6 (69.3–84.6)
Grade 3	2	5.6 (0.7–18.7)	6	4.8 (1.8–10.2)
Redness	15	41.7 (25.5–59.2)	47	37.6 (29.1–46.7)
Grade 3	0	0.0 (0.0–9.7)	1	0.8 (0.0–4.4)
Swelling	7	19.4 (8.2–36.0)	30	24.0 (16.8–32.5)
Grade 3	0	0.0 (0.0–9.7)	0	0.0 (0.0–2.9)
*Solicited general* adverse events				
Fatigue	8	22.2 (10.1–39.2)	38	30.4 (22.5–39.3)
Grade 3	0	0.0 (0.0–9.7)	3	2.4 (0.5–6.9)
Gastrointestinal symptoms	1	2.8 (0.1–14.5)	11	8.8 (4.5–15.2)
Grade 3	0	0.0 (0.0–9.7)	2	1.6 (0.2–5.7)
Headache	8	22.2 (10.1–39.2)	40	32.0 (23.9–40.9)
Grade 3	0	0.0 (0.0–9.7)	3	2.4 (0.5–6.9)
Fever (≥37.5°C)	1	2.8 (0.1–14.5)	3	2.4 (0.5–6.9)
Grade 3	0	0.0 (0.0–9.7)	0	0.0 (0.0–2.9)
	N = 37		N = 128	
Unsolicited adverse events				
Any adverse event	10	27.0 (13.8–44.1)	33	25.8 (18.5–34.3)
Related to vaccination	1	2.7 (0.1–14.2)	5	3.9 (1.3–8.9)
Grade 3	2	5.4 (0.7–18.2)	3	2.3 (0.5–6.7)
Large swelling reactions	0	0.0 (0.0–9.5)	0	0.0 (0.0–2.8)
Serious adverse events	0	0.0 (0.0–9.5)	0	0.0 (0.0–2.8)

Td group, participants receiving Td as first booster dose in the primary study and Tdap asdecennialboosterdose(secondboosterdose) in the current study; Tdap group, participants receiving Tdap booster doses 10 years apart; n (%), number (percentage) of participantsreportingtheadverseeventatleast once; CI, confidence interval; N,numberofparticipantswithdocumenteddoses (for solicited adverse events) or administered doses (for unsolicited adverse events).

Note:Grade3 wasdefinedasdiameter > 50 mm (for redness and swelling), temperature >39.0°C (for fever) and as preventing normal activity for all other adverse events.

Gastrointestinal symptoms include nausea, vomiting, diarrhea and/or abdominal pain.

Large swelling reactions were defined as swelling with a diameter >100 mm, noticeable diffuse swelling or noticeable increase of limb circumference.

During the 31-day post-vaccination period, at least one unsolicited AE was reported for 27.0% participants in the Td group and 25.8% participants in the Tdap group, with headache being the most frequently reported AE in each group (by 13.5% adults in the Td group and 9.4% of adults in the Tdap group). At least one grade 3 unsolicited AE was reported in 2 (5.4%) participants in the Td group and 3 (2.3%) participants in the Tdap group. One (2.7%) unsolicited AE (myalgia) in the Td group and 5 (3.9%) in the Tdap group (influenza like illness, injection site pruritus, pain in extremity, paraesthesia, general and maculo-papular rash) were assessed by the investigators as causally related to vaccination. Medically-attended unsolicited AEs were reported in 2 (5.4%) participants in the Td group and 6 (4.7%) participants in the Tdap group.

None of the AEs led to premature withdrawals from the study. No large swelling reactions and no serious AEs were reported in this study and the incidence of all AEs was comparable between groups.

No hospitalizations, pregnancies or serious AEs (SAEs) were reported during the study.

## Discussion

This study demonstrated the non-inferiority of a second decennial Tdap booster dose to a single Tdap booster dose given to a population previously boosted with Td with respect to the immune response against diphtheria and tetanus antigens and to a 3-dose series of DTaP administered during infancy with respect to immune responses to the pertussis vaccine components.

In the absence of established correlates of protection for pertussis, an immuno-bridging approach was used in previous studies to assess immune responses to pertussis antigens, by extrapolating the efficacy of DTaP against pertussis as demonstrated in infants to an older age group.^27^^,^^29^^,^^31^ Therefore, antibody GMCs induced by a second decennial Tdap booster dose in our study were compared to those elicited in infants receiving DTaP^32^ in a study which also demonstrated a 88.7% efficacy against pertussis.^30^ As the non-inferiority of antibody levels for pertussis antigens in our study to those elicited by the 3-dose DTaP infant series was demonstrated, the administration of a second booster Tdap dose is expected to induce an immune response consistent with protection against disease.

Antibody persistence observed in our study at 10 years after the first booster vaccination with either Td or Tdap was comparable between the 2 groups for diphtheria and tetanus. However, seropositivity rates and antibody GMCs for pertussis antigens tended to be higher in the group having previously received Tdap than in individuals previously boosted with Td. The results in the Tdap group are consistent with persistence data observed in a decennial Tdap trial conducted in Australian adults, for whom seropositivity rates ranged from 94.7% to 99.3% for FHA, PRN and tetanus and were 85.6% for PT, before a second Tdap booster dose. However, seroprotective anti-diphtheria antibody concentrations were observed in only 89.4% of participants,^27^ while in the current study all adults were seroprotected prior to the second booster dose. In the same study, pre-booster immune responses to pertussis tended to be higher in participants receiving 2 sequential Tdap doses compared to those receiving separate Td and pa vaccination followed by a Tdap dose 10 years later,^27^ similarly to our observations. Nevertheless, as in our study, ≥99.3% of Australian adults had seroprotective/seropositive antibody levels against each Tdap antigen following the second Tdap booster dose.^27^ Our results are also in line with those from a study conducted in young adults in Finland vaccinated with a second booster dose of Tdap, 10 years after prior administration of the vaccine.^28^ Of note, the vaccine formulation approved for use in the US has a lower aluminum adjuvant content (≤0.39 mg/dose) than the one licensed in the rest of the world (0.5 mg/dose), although differences in the aluminum content were previously shown not to impact persistence of immune responses to Tdap antigens at 10 years post-boosting.^33^

The booster response rates to diphtheria and tetanus antigens were similar in the Td and Tdap groups, although relatively low values (40.0–60.0%) were observed, regardless of the number of Tdap booster doses received. The high antibody levels at baseline documented in our study might account for this lower-than-anticipated response, as all participants had pre-booster antibody concentrations ≥0.1 IU/mL for both antigens, compared to other studies in which seropositivity levels were 82.4–89.4% for diphtheria and 85.7–97.3% for tetanus.^27^^,^^28^ Indeed, when calculating the booster response adjusted for high baseline concentrations, higher values were found in both groups, with at least 59.2% of participants exhibiting a booster response for diphtheria and 82.9% for tetanus. However, alternative booster responses differed between groups for diphtheria (but not tetanus), with higher point estimates observed in the Td compared with the Tdap group, although this difference was not statistically significant, as shown by overlapping 95% CIs. Moreover, the relatively low booster responses and the difference between groups observed for diphtheria are not likely to have any clinical significance, since all participants in the Tdap group had seroprotective levels for both diphtheria and tetanus following the administration of the second Tdap dose, in line with post-booster results observed in all age groups following Tdap vaccination.^34^

Booster responses to pertussis antigens were similar in the two groups and more robust for PT and FHA (>90.0%) than for PRN (>68.0%). In the decennial study conducted in Australia, a second dose of Tdap elicited a booster response rate for PT (97.4%) and PRN (69.7%) similar to that found in our study, but a lower response rate for FHA (78.3%).^27^ In a decennial study conducted in Finland, booster response rates were similar for PT (98.6%) and FHA (97.3%), but higher for PRN (93.2%)^28^ as compared to those observed in this study. The booster response rates in our study might have been impacted by the fact that the vast majority of the participants in each group already had pre-booster antibody levels above the used threshold, a phenomenon which has been previously noted for booster response to PRN in individuals with high pre-vaccination anti-PRN antibody concentrations.^27^ Nevertheless, our results confirm previous reports of a robust post-vaccination response against pertussis following administration of a Tdap booster dose, although several different, often arbitrary, definitions were used in other studies for booster response.^34^ A slightly decreased post-booster response for PT was previously noted for Tdap formulations with a lower aluminum content compared to formulations containing 0.5 mg/dose, but differences between either 0.5 mg or 0.3 mg aluminum-containing Tdap were not associated with clinically important differences in protection against any of the vaccine antigens.^33-35^ The overall booster response rate observed for participants in the Tdap group in our study was similar to that reported for adults and adolescents receiving the vaccine containing 0.5 mg of aluminum per dose and higher than those receiving lower-content formulations.^35^ However, in the latter study, booster response was defined as a ≥2- (for diphtheria and tetanus) or ≥4- (for pertussis) fold increase from pre-vaccination levels in initially seropositive participants and post-vaccination levels of ≥4 times the assay cut-off for initially seronegative participants and a different assay cut-off was used.^35^

A second dose of Tdap vaccine administered to the Tdap group had a comparable safety profile to that of a first dose of Tdap administered to the Td group. Large injection-site reactions, reported for DTaP, diphtheria or tetanus vaccines,^28^ were not observed in any of the study groups. Moreover, no SAEs were reported in this study.

Immunity against tetanus and diphtheria may last longer than the recommended 10-year booster dose interval,^36^ but protection against pertussis following Tdap vaccination wanes more rapidly than previously anticipated.^25^ The current recommendations on the use of a one-time only Tdap boosting might be putting the generation of US adolescents and adults at high risk of developing pertussis. With no stand-alone pertussis vaccine available at the moment, additional boosting at 10-year intervals could help maintain immunity against the disease and guarantee better protection during pertussis outbreaks. A recent study assessed the risk of pertussis of adolescents vaccinated with Tdap during two pertussis outbreaks in California in 2010 and 2014 and outlined the low degree of protection at 2–3 years following vaccination, suggesting that a pertussis-containing booster vaccination should be given in anticipation of an outbreak rather than as routine vaccination.^25^ Our study is among the first to assess immunogenicity of decennial Tdap boosting in the US. Only one other Tdap vaccine (*Adacel*, Sanofi Pasteur) is licensed for use as a booster dose in adolescents and adults and showed good immunogenicity and safety in adults after the administration of a repeat dose, at 10 years after previous Tdap administration.^37^ Results from both studies are paving the way for the implementation of an additional booster dose in US adolescents and/or adults, although vaccination strategies will have to be tailored to take into account current recommendations for special and at-risk populations.

The study was designed with sufficient power to assess the co-primary confirmatory objectives. Despite the lower-than-expected sample size for the extension study, a sensitivity analysis conducted to evaluate the robustness of GMC results with respect to dropout from the primary study demonstrated no apparent bias related to dropout. However, generalization of results should be performed with caution, due to the limited sample size at the pre-vaccination time point and differences in the aluminum content if comparisons are made with Tdap formulations licensed outside US. Moreover, booster responses for pertussis antigens were defined using the cut-offs of newly-validated assays and therefore they can differ significantly from those already reported in the literature.

**Figure 2.**
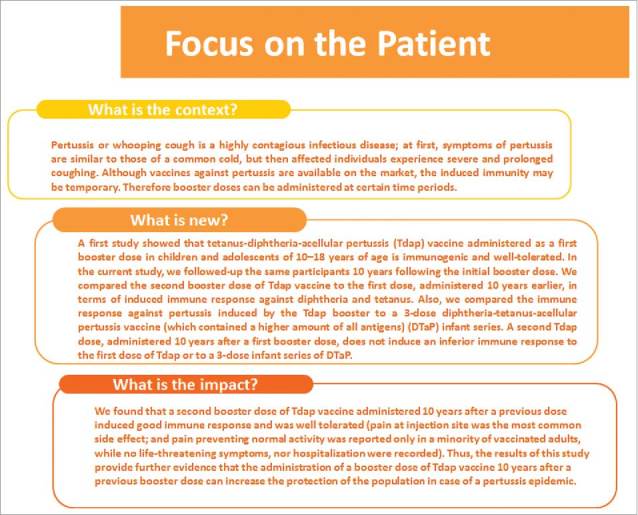
Focus on Patient section.

A lay language graphical summary contextualizing the results and potential clinical research relevance and impact of our study is displayed in the Focus on Patient Section (Fig. 2).

## Conclusions

Immune responses to a second decennial Tdap booster dose were non-inferior to a single Tdap booster dose given to a population previously boosted with Td for diphtheria and tetanus and to a 3-dose series of DTaP administered during infancy for pertussis antigens. The Tdap booster dose administered 10 years following vaccination against diphtheria and tetanus (Td) or previous Tdap dose was shown to be immunogenic and well-tolerated in young adults aged 19–30 years. These results support the administration of a second decennial booster Tdap dose to increase protection and prolong immunity for diphtheria, tetanus and pertussis.

## Material and methods

### Study design and participants

This phase III, open-label, non-randomized, follow-up study (NCT01738477) with two parallel groups took place between January 2013 and April 2014 in 20 centers in the US. The study enrolled healthy individuals aged 19–30 years who had correctly received a booster of either a Tdap or the control Td vaccine 10 years (±300 days) before. In the primary study (NCT00109330), participants aged 10–18 years received 3 different lots of Tdap or the control vaccine Td,^29^ while in the extension study, all enrolled participants received a Tdap dose (Fig. 1).

Exclusion criteria at enrolment included vaccination against or history of diphtheria, tetanus or pertussis disease since the last dose received in the primary study, use of any vaccine not foreseen by the study protocol (except for influenza) 30 days before and during the study period, and receipt of immune modifying drugs within six months preceding the booster vaccine dose. Pregnant or lactating women were excluded from enrolment.

This study was conducted in an open-label, non-randomized manner, since all the participants received a single dose of Tdap. The 0.5 ml dose was administered intramuscularly in the deltoid muscle of the non-dominant arm and contained 2.5 Lf diphtheria toxoid, 5 Lf tetanus toxoid, 8 µg PT, 8 µg FHA, 2.5 µg PRN, Al(OH)_3_ ≤ 0.39 mg and NaCl.

The study was performed in accordance with the Good Clinical Practice guidelines and the Declaration of Helsinki. Written informed consent was obtained from each participant prior to enrolment. The study protocol and informed consent form were reviewed and approved by an Independent Ethics Committee or Institutional Review Board at each center. The trial was registered with www.ClinicalTrials.gov (NCT01738477) and a protocol summary is available from http://www.gsk-clinicalstudyregister.com (study ID 116570).

### Objectives

The first co-primary objective assessed the non-inferiority of a second Tdap booster dose to a first dose of Tdap vaccine, with respect to immune response to diphtheria and tetanus antigens at one month post-vaccination. The second co-primary objective evaluated the non-inferiority of a second booster dose of Tdap to a three-dose series of DTaP vaccine administered in infancy in a German household contact study which demonstrated vaccine efficacy against pertussis,^30^ with respect to immune responses against PT, FHA and PRN. The co-primary objectives were assessed in a hierarchical manner. Secondary objectives included the evaluation of antibody persistence, immune responses to Tdap antigens in both study groups and comparing the safety of a second dose to a first Tdap dose.

### Immunogenicity assessment

Blood samples of approximately 5 ml were collected from all participants before (pre-booster) and one month after vaccination (post-booster). Anti-diphtheria, anti-tetanus, anti-PT, anti-FHA and anti-PRN antibody concentrations were assessed using a recently validated, in-house enzyme-linked immunosorbent assay (ELISA) at GSK, Belgium; therefore, the assay cut-offs for the assessments were different from those used in the primary study. The serological assays determined immunoglobulin G (IgG) antibodies against each vaccine component and were quantified using purified diphtheria toxoid extracted from *Corynebacterium diphtheriae*, tetanus toxoid extracted from *Clostridium tetani*, FHA, PT and PRN antigens extracted from *B. pertussis* culture in virulence phase I as coating. For each assay, the antigen was coated onto a 96-well microplate. After a washing and a blocking step, the diluted serum samples, the controls and each standard were incubated on the coated plate. The microplate was washed and mouse horseradish peroxidase-conjugated anti-human IgG monoclonal antibodies were added. After incubation, unbound antibodies were removed by washing and the microplate was incubated with tetra-methyl-benzidine, to reveal enzyme activity. The color reaction was stopped by the addition of sulfuric acid and the resulting yellow color was measured spectrophotometrically. Antibody concentrations were calculated from a reference standard curve calibrated against the World Health Organization International Standard (NIBSC 06/140) using a 4-parameter logistic fitting algorithm and expressed in IU/ml. The new ELISAs were validated following the Food and Drug Administration's guidance for methods validation for drugs and biologics^38^ and their comparability to previous assays was assessed in extensive bridging experiments assessing seropositivity rates and GMCs. The new assay cut-offs were 0.057 IU/ml and 0.043 IU/ml for antibody concentrations for diphtheria and tetanus, respectively. Samples with pre-vaccination anti-diphtheria antibody concentrations <0.1 IU/ml were also tested by a Vero cell neutralization assay, with a cut-off of 0.004 IU/ml. Antibody concentrations ≥0.1 IU/ml for diphtheria and tetanus were considered to provide a conservative estimate of protection.^39^^,^^40^ As no correlate of protection is defined for pertussis antigens,^41^^,^^42^ the newly validated ELISA cut-offs of 2.693 IU/ml for PT, 2.046 IU/ml for FHA, and 2.187 IU/ml for PRN antibody concentrations were used to define seropositivity.

### Safety assessment

AEs were recorded by participants using diary cards, which were returned at the following visit. The occurrence of all solicited local and general AEs, as well as that of large swelling reactions, was documented within 4 days (Day 0–3) post-vaccination. Unsolicited AEs, medically-attended AEs and serious AEs were collected within the 31-day (Day 0–30) period following vaccination. All solicited and unsolicited AEs were graded by severity on a scale from 1 (mild) to 3 (severe). All solicited local (injection site) reactions were considered to be causally related to vaccination, while the causality of all other AEs was assessed by the investigators.

### Statistical analyses

With 100 participants in the Td and 300 participants in the Tdap group, the overall power to demonstrate both co-primary objectives simultaneously was 97%. Considering that 20% of participants might not be evaluable, blood samples needed to be taken from a minimum of 125 individuals in the Td group and 375 in the Tdap group, assuming a comparable group allocation ratio to that of the primary study.

Non-inferiority was demonstrated if, at one month after vaccination, the LLs of the 95% CI on the difference of the seroprotection rates (Tdap minus Td group) for anti-diphtheria/tetanus antibody concentrations were ≥-10% or if one month after vaccination, the LLs of the 95% CI on the anti-PT, anti-FHA and anti-PRN antibody GMC ratios (Tdap group divided by comparator group from the German household contact study) were ≥0.67. Seropositivity/seroprotection rates and antibody GMCs pre- and one month post-vaccination and booster response rates at one month post-vaccination were calculated with exact 95% CIs.

GMCs were computed by taking the anti-log of the mean of the log concentration/titer transformations. Antibody concentrations below the cut-off of the assays were given an arbitrary value of half the cut-off. The associated CIs for between-group GMC ratios were derived using the method proposed by G.Y. Zou and A. Donner,^43^ in order to account heterogeneity of variance between this study and the DTaP German household contact study.

An analysis of persistence was carried out in order to evaluate the robustness of the results with respect to dropout, by using a repeated generalized linear model. This model used results from the post-vaccination visit in the parent study and pre-booster results of the extension.

The percentage of participants reporting (serious) AEs were tabulated with exact 95% CIs.

The immunogenicity analysis was performed on the ATP cohort for immunogenicity, which included all vaccinated participants who complied with the study procedures and had available immunogenicity data. The safety analysis was performed on the total vaccinated cohort, which included all participants receiving a Tdap dose.

All analyses were carried out using SAS version 9.22.

*Boostrix* and *Infanrix* are trade marks of the GSK group of companies. *Adacel* is a trade mark of Sanofi Pasteur.

## Supplementary Material

KHVI_A_1460292_supplemental.docx

## Abbreviations

AE: adverse event
ATP: according-to-protocol
CI: confidence interval
DTaP: diphtheria-tetanus-acellular pertussis vaccine
ELISA: enzyme-linked immunosorbent assay
FHA: filamentous hemagglutinin
GMC: geometric mean concentration
IU: International Units
LL: lower limit
PRN: pertactin
PT: pertussis toxoid
S−: seronegative
S+: seropositive
SAE: serious adverse event
SD: standard deviation
Td: tetanus-diphtheria vaccine
Tdap: tetanus-diphtheria-acellular pertussis vaccine
US: United States.
