# Supplementary material for: Immunogenicity and safety of a second booster dose of an acellular pertussis vaccine combined with reduced antigen content diphtheria-tetanus toxoids 10 years after a first booster in adolescence: An open, phase III, non-randomized, multi-center study
Source: Hum Vaccin Immunother. 2018 May 10;14(8):1977–86. doi: 10.1080/21645515.2018.1460292 (PMC6149833; doi:10.1080/21645515.2018.1460292)
Supplement: KHVI_A_1460292_supplemental.docx [file khvi-14-08-1460292-s001.docx]

**Online supplement. Sensitivity analysis**

An analysis of persistence was carried out in order to evaluate the robustness of the results with respect to dropout, by using a repeated generalized linear model. This model used results from post-vaccination visit of the primary study (NCT00109330) and pre-booster results of the current study (NCT01738477). The analyses were performed on the ATP cohort of immunogenicity of the primary and current study, respectively.

Serology results below the assay cut-off values were considered as left censored at the assay cut-off. The model included the fixed group effect, the fixed effect of time since last vaccination, the random intercept effect for all serology, random slope effect for diphtheria and tetanus, and interaction in slope for pertussis antigens. The results of the analysis are presented in **Table S1**.

**Table S1.** Observed and modelled antibody geometric mean concentrations and 95% confidence intervals for Tdap antigens (according-to-protocol cohort for immunogenicity adapted for each timepoint)

|  | Td group | | |  | Tdap group | | |
| --- | --- | --- | --- | --- | --- | --- | --- |
|  | N | Observed GMC | Modelled GMC |  | N | Observed | Modelled |
| *Diphtheria* |  |  |  |  |  |  |  |
| Post-booster, initial study | 998 | 14.1 (13.4–15.0) | 14.1 (13.3–14.9) |  | 2975 | 7.3 (7.1–7.6) | 7.3 (7.1–7.6) |
| Pre-booster, current study |  |  | 2.4 (2.0–3.0) |  |  |  | 1.3 (1.0–1.6) |
| *Tetanus* |  |  |  |  |  |  |  |
| Post-booster, initial study | 998 | 20.7 (19.7–21.7) | 20.6 (19.6–21.6) |  | 2976 | 15.8 (15.3–16.2) | 15.8 (15.3–16.3) |
| Pre-booster, current study |  |  | 2.2 (1.9–2.5) |  |  |  | 1.7 (1.4–1.9) |
| *Pertussis toxoid* |  |  |  |  |  |  |  |
| Post-booster, initial study | 986 | 9.9 (9.1–10.7) | 9.1 (6.2–13.4) |  | 2939 | 86.8 (83.9–89.9) | 98.4 (80.7–120.0) |
| Pre-booster, current study |  |  | 5.3 (3.6–7.7) |  |  |  | 10.2 (8.4–12.4) |
| *Filamentous hemagglutinin* |  |  |  |  |  |  |  |
| Post-booster, initial study | 995 | 38.8 (35.9–41.9) | 46.3 (33.4–64.0) |  | 2977 | 614.5 (596.0–633.5) | 610.6 (510.3–730.5) |
| Pre-booster, current study |  |  | 21.8 (15.8–30.0) |  |  |  | 37.0 (31.0–44.1) |
| *Pertactin* |  |  |  |  |  |  |  |
| Post-booster, initial study | 997 | 11.8 (10.9–12.9) | 15.0 (9.0–24.9) |  | 2976 | 470.9 (447.2–495.8) | 547.6 (417.5–718.3) |
| Pre-booster, current study |  |  | 27.8 (17.1–45.4) |  |  |  | 71.8 (54.9–94.0) |

Td group, participants receiving Td as first booster dose in the primary study and Tdap as decennial booster dose (second booster dose) in the current study; Tdap group, participants receiving Tdap booster doses 10 years apart; N, number of participants with available results at 1 month post-booster dose in the primary study.
